# Effects of a 3-Week Inpatient Multidisciplinary Body Weight Reduction Program on Body Composition and Physical Capabilities in Adolescents and Adults With Obesity

**DOI:** 10.3389/fnut.2022.840018

**Published:** 2022-03-31

**Authors:** Stefano Lazzer, Mattia D’Alleva, Filippo Vaccari, Gabriella Tringali, Roberta De Micheli, Alessandro Sartorio

**Affiliations:** ^1^Department of Medicine, University of Udine, Udine, Italy; ^2^School of Sport Sciences, University of Udine, Udine, Italy; ^3^Experimental Laboratory for Auxo-Endocrinological Research, Istituto Auxologico Italiano, Scientific Institute for Hospitalization and Care (IRCCS), Piancavallo, Italy; ^4^Division of Auxology, Istituto Auxologico Italiano, Scientific Institute for Hospitalization and Care (IRCCS), Piancavallo, Italy

**Keywords:** physical capabilities, body composition, adolescents, adults, obesity, physical activity

## Abstract

**Background:**

The aim of the present study was to examine the short-term changes in body composition and physical capabilities in subjects with obesity during a multidisciplinary inpatient body weight reduction program (BWRP).

**Methods:**

One hundred thirty-nine adolescents (56 boys and 83 girls; BMI: 37.1 ± 6.5 kg/m^2^; Fat Mass, FM: 45.3 ± 7.2%) and 71 adults (27 males and 44 females; BMI: 44 ± 4.7 kg/m^2^; FM: 51.4 ± 4.7%) followed a 3-week inpatient BWRP consisting of regular physical activity, moderate energy restriction, nutritional education and psychological counseling. Before (T0) and after the end of the BWRP (T21), body composition was assessed with an impedancemeter, lower limb muscle power with Margaria Stair Climbing Test (SCT), lower limb functionality with Short Physical Performance Battery (SPPB), and the capacity of performing activity of daily living (ADL) with Physical Performance Test (PPT).

**Results:**

At T21, obese adolescents showed a 4% reduction in body mass (BM) (*p* < 0.001), associated with a FM reduction in boys (−10%) and girls (−6%) (*p* < 0.001) and with a 3% reduction in fat-free mass (FFM) recorded only in boys (*p* = 0.013). Obese adults showed a 5% BM reduction (*p* < 0.001), associated with a 2% FFM and 9% FM reduction (*p* < 0.001) in males, and 7% FM reduction in females (*p* < 0.001). Regarding physical capabilities, at T21 in obese adolescents, PPT score increased by 4% (*p* < 0.001), SCT decreased by −5% (boys) and −7% (girls) (*p* < 0.001), while SPPB score did not change significantly. In obese adults at T21, PPT score increased by 9% (*p* < 0.001), SCT decreased by −16% (*p* < 0.001) only in females, and SPPB score increased by 7% (males) and 10% (females) (*p* < 0.01).

**Conclusion:**

In conclusion, moderate energy restriction and regular physical activity determine a 4-5% BM reduction during a 3-week inpatient BWRP, improve physical capabilities and induce beneficial changes in body composition in adolescents and adults with obesity.

**Trial registration:**

This study was approved by the Ethical Committee of the Istituto Auxologico Italiano (Milan, Italy; research code: 01C124; acronym: PRORIPONATFIS). Registered 11 November 2020 - Retrospectively registered.

## Introduction

Over the past two decades, obesity has tripled in both children and adults in industrialized countries (1, 2), mainly due to the excess food-intake combined with an increase in time spent in sedentary activities, leading to a decrease in physical activity level (3–5). Excessive levels of body fat is associated with major health consequences associated with obesity, including hypertension (6–8), type 2 diabetes, and cardiovascular disease (9). In addition, reduced physical activity levels in adolescents and adults with obesity is reported to lead to a lower aerobic and anaerobic capacities, and lower limb muscle power output than their normal-weight counterparts (10–12). Consequently, obese subjects with high values of fat mass and poor physical abilities, usually suffer from lower limb functionality, higher perceived difficulty in performing physical exercise and activities of daily living (ADL) (13–16). Nevertheless, in obese subjects, short-term multidisciplinary inpatient body weight-reduction programs (BWRP) entailing moderate-intensity physical training, energy-restricted diets, and changes in food and behavioral habits have been reported to promote weight loss, reduce fat mass (FM) (17–19) and improve physical capacities. The improvements in body composition and physical capacities is reported to exert a positive effects on metabolic and cardiovascular risk profiles (20, 21), as well on both lower limb muscle power and functionality (17–19) and on performing ADL (13). However, the presence of a lower limb function and ability to perform ADL is often overlooked in adolescents and adults with obesity (22), even if their poor physical abilities actually worsen their quality of life, as it happens elderly obese (22).

Therefore, the aim of the present study was to determine whether a short-term (3-week) multidisciplinary BWRP entailing physical activity, moderate energy restriction, nutrition education, and psychological counseling, can positively affect body composition, lower limb muscle power and functionality, capacity of performing ADL in adolescents and adults with obesity.

## Materials and Methods

### Subjects

One hundred thirty-nine adolescents (56 boys and 83 girls; age range: 13–17 years) and 71 adults (27 males and 44 females; age range: 35–68 years) with obesity, participated in this study. Among adolescents, BMIs for gender and chronological age were above the 99th percentile (23), while for adults the BMIs were above 35 kg/m^2^. Subjects were recruited as inpatients from the Division of Auxology (subjects aged <18 year) and from the Division of Metabolic Diseases (subjects aged >18 years), Istituto Auxologico Italiano, IRCCS, Piancavallo (VB), Italy. Before admission to the hospital for BWRP, none of the subjects had engaged in structured physical activity (i.e., regular activity of more than 60 min/week) evaluated with the validated International Physical Activity Questionnaire Short Form (IPAQ-SF) (24). All subjects had a complete medical history and physical examination. None of the adolescents or adults with obesity had signs or symptoms indicative of serious cardiovascular, respiratory, or orthopedic disease that could significantly interfere with the functional test used in the study.

### Study Protocol

The study was approved by the Ethical Committee of the Istituto Auxologico Italiano (Milan, Italy; research code: 01C124; acronym: PRORIPONATFIS) and was in accordance with the Declaration of Helsinki 1975, as revised in 2008. For adolescents, the protocol was explained to parents and written informed consent was obtained from parents or legal representatives. Patients aged ≥18 gave written informed consent to participate in the study. Patients were hospitalized for a period of 3 weeks in the Division of Auxology (patients <18 years) or in the Division of Metabolic Diseases (patients >18 years), Istituto Auxologico Italiano, IRCCS, Piancavallo (VB). They followed a 3-week personalized BWRP consisting of moderate energy restriction, physical activity, nutrition education, and psychological counseling. Full testing sessions were conducted at the beginning (T0) and at completion of the 3-week BWRP (T21). Testing session included assessment of anthropometric characteristics, body composition, blood pressure (BP), lower limb muscle power, lower limb functionality, and ability to perform ADL (see below for detailed description).

#### Physical Activity

The physical activity program consisted of five training days per week, under the supervision of a physical trainer. Each training session included: (i) 45–60 min per day of aerobic activities (walking on a treadmill or cycling on an ergometer) under heart rate monitoring (HR) and medical supervision and (ii) 5–7 min of stretching before and after training. The intensity of aerobic activities was set at heart rate (HR) corresponding to 60 and 80% of the individual maximal HR estimated as 220-age (year). The research assistant and the physical trainers verified that each subject participated in each training session, performed the exercises correctly, and completed at least 95% of the exercise session and program. In addition, subjects had 1 h/day of aerobic leisure activities at the institution on Saturday and Sunday.

#### Diet and Nutrition Education

A Mediterranean diet was prescribed based on the initial basal metabolic rate test and physical activity level for each patients, and the amount of energy to be given with diet was calculated by subtracting approximately ∼25% from the estimated daily energy expenditure. In terms of macronutrients, the diet contained 21% proteins, 53% carbohydrates, and 26% lipids. The diet composition was formulated according to the Italian recommended daily allowance (25). Each patient was free to choose foods from a heterogeneous daily menu, although five daily servings of fruits and vegetables were mandatory. Foods to which the patient reported allergic reactions were eliminated from the menu. A fluid intake of at least 1.5 L/day was encouraged. In addition, the dietitian team checked that each subject had eaten every meal. On each day of the BWRP, the patients had dietetics classes consisting of lectures, demonstrations, and group discussions with and without a supervisor.

#### Psychological Counseling

Cognitive-behavioral therapy strategies, such as stimulus control procedures, problem-solving and stress management training, development of healthy eating habits, assertiveness and social skills training, cognitive restructuring of negative maladaptive thoughts, and relapse prevention training, were chosen for the psychological sessions, which were conducted by a clinical psychologist 2–3 times per week in individual or group sessions as previously reported (26). When possible (1 day per week), additional sessions were also conducted with parents of the obese adolescents aimed at improving motivation for lifestyle change and interpersonal communication.

### Measurements

#### Physical Characteristics and Body Composition

Medical history was obtained and a baseline physical examination was performed. Stature and body mass (BM) were measured using a Harpenden stadiometer (Holtain Ltd., United Kingdom), and an electronic scale (Selus, Italy), respectively, with the subject wearing only light underwear. BMI (kg/m^2^) was calculated. The standard deviation score (SDS) of BMI-SDS was calculated using the LMS method (23) on Italian reference values for children and adolescents (27). Body composition was measured using a multifrequency tetrapolar impedancemeter (BIA, Human-IM Scan, DS-Medigroup, Milan, Italy) with a delivered current of 800 μA at a frequency of 50 kHz. To reduce measurement errors, care was taken to standardize the variables that affect the validity, reproducibility and precision of the measurement. The measurements were performed according to the method of Lukaski et al. (28) (after 20 min of rest in the supine position with arms and legs relaxed and without contact with other parts of the body) and under strictly controlled conditions according to NIH guidelines (29). Before measurements, technical accuracy has been validated by an external parallel circuit containing a high-precision resistor and capacitor. Low-impedance electrodes were used for reliable and accurate assessment of the raw bioimpedance parameters (e.g., R, Xc, and phase angle) (30). The within-day coefficient of variation for three repeated assessments of FFM in obese subjects (with repositioning of electrodes) has been previously assessed in our laboratory (2.4%).

All females were studied outside of the menstrual period in order to avoid any possible influence on fluid retention, as suggested by the NIH guidelines (29). For the adolescents, Fat-Free Mass (FFM) was calculated using the prediction equation previously developed by our group against DEXA (31):


$$FFM\left(kg\right)=0.87\cdot \text{ZI}\left({\text{cm}}^{2}\cdot \Omega \right)+3\cdot 1(\mathrm{adjusted}\mathrm{coefficient}ofvariation=0.91)$$

where, ZI is the impedance index calculated as stature (cm^2^) divided by whole-body impedance (Z) at 50 kHz (Ω). For the adults, we used the equation developed by Gray et al. (32):


$$F\,F\,M\,\,\left(k\,g\right)=0.00139\cdot \text{stature}\,\left({\text{cm}}^{2}\right)\,-\,0.0801\cdot \text{Z}\,\left(\Omega \right)+0.\mathrm{l87}\cdot \text{BM}\,\left(\text{kg}\right)$$

FM (kg) was derived as the difference between BM (kg) and FFM (kg).

BP measurements were taken after the participants have rested in the sitting position for at least 5 min, the average of 2 BP readings was used for analysis.

#### Lower Limb Muscle Power

The Stairs Climbing Test (SCT) is a well-standardized procedure for measuring maximal anaerobic power in adolescents and adults with obesity (20, 33). Prior to administering the test, 2–3 practise trials were scheduled to allow subjects to gain sufficient confidence with the technique. Briefly, subjects were asked to climb an ordinary stair at the highest possible speed, according to their abilities. The stairs consisted of 13 steps of 15.3 cm each, so that a total vertical distance of 1.99 m was covered. An experimenter measured the time taken to complete the test using a digital stopwatch. SCT repeatability in obese subjects has been previously evaluated in our laboratory and the coefficient of variation between measurements was found to be lower than 5% (20).

### Short Physical Performance Battery

A Short Physical Performance Battery (SPPB) (34, 35) was administered. The SPPB consists of the following three parts: (i) tests of standing balance, included semi-tandem position, side-by-side stands and tandem position (each held for 10 s), (ii) walking a 4 m distance at normal gait speed, and (iii) rising from a chair and returning to the seated position five times. Scores for each item ranged from 0 to 4, for a maximum of 12 points. Performance categories were created for each set of performance measures to allow for analyses that included those unable to perform a task. The three tests of standing balance were considered hierarchical in difficulty by assigning a single score from 0 to 4 for standing balance (35). For 4 m walking and repeated chair stands, a score of 0 was assigned to those who could not complete the task. Those who were able to complete the task were assigned scores from 1 up to 4, corresponding to quartiles of time required for the task, with the fastest times scored as 4 (35). Higher scores were associated with better lower limb functionality (34).

#### Physical Performance Test

The ability to perform ADL was assessed using the Physical Performance Test (PPT) (36). The PPT test used in the present study includes 7 standardized tasks [(i) walk 15.2 m, (ii) put on and take off a coat, (iii) pick up a coin, (iv) lift a book, (v) simulate the act of eating, (vi) perform a 360° turn, and (vii) write a sentence]. The score for each item ranged from 0 to 4, with 0 corresponding to “unable to do” and 4 corresponding to “most able or quickest” (36). The maximum score was 28, and participants were classified as mildly to moderately frail if they scored between 19 and 24 (37).

### Statistical Analyses

Statistical analyses were performed using Graph Pad Prism version 9.1.0-2021 software (GraphPad Software, Inc., San Diego, CA, United States) with a significance set at *p* < 0.05. All results were expressed as mean and standard deviation (SD). Normal distribution of the data was tested using the Kolmogorov–Smirnov test. The effects of gender, time, and the interaction between these variables on physical characteristics, body composition, lower limb muscle power, lower limb functionality and ability to perform ADL were tested using General Linear Model repeated measures. When significant differences were found, a Bonferroni *post hoc* test was evaluated implementing multiple comparisons. Relationships between the different factors were examined using Pearson or Spearman product–moment correlation coefficient.

## Results

### Effects of the 3-Week BWRP in Adolescents

#### Anthropometric Characteristics and Body Composition

At T0, age, BM, BMI, and BMI-SDS were not significantly different between boys and girls, whereas stature was ∼5% higher in boys than girls (*p* < 0.001, Table 1). At the end of BWRP (T21), BM and BMI decreased by ∼4% (*p* < 0.001, Table 1) in both genders, a greater reduction (∼7%, *p* < 0.001, Table 1) being observed in BMI-SDS in both genders.

**TABLE 1:** Physical characteristics of adolescents with obesity before (T0) and after (T21) the 3-week BWRP.

	Boys (*n*: 56)		Girls (*n*: 83)		*P*		
	T0	T21	T0	T21	G	T	G × T
Age (y)	14.9 ± 2.0		15.0 ± 2.1		0.841		
Stature (m)	1.68 ± 0.11		1.60 ± 0.07		0.001		
Body mass (kg)	103.3 ± 22.7	98.7 ± 21.8	96.8 ± 21.3	92.7 ± 20.2	0.095	0.001	0.154
BMI (kg/m^2^)	36.4 ± 5.7	34.8 ± 5.6	37.5 ± 7.3	35.9 ± 6.9	0.338	0.001	0.906
BMI (SDS)	2.9 ± 0.6	2.7 ± 0.6	2.9 ± 0.6	2.4 ± 0.6	0.637	0.001	0.028
Fat-free mass (kg)	59.3 ± 11.6	57.4 ± 13.3	48.6 ± 7.6	47.7 ± 7.8	0.001	0.003	0.226
Fat-free mass (%)	57.8 ± 5.5	59.5 ± 6.7	51.1 ± 6.6	52.5 ± 7.2	0.001	0.001	0.635
Fat Mass (kg)	44.0 ± 13.4	40.1 ± 14.5	48.0 ± 16.1	44.8 ± 15.3	0.091	0.001	0.345
Fat Mass (%)	42.2 ± 7.7	40.5 ± 6.7	48.4 ± 6.7	47.4 ± 7.3	0.001	0.002	0.503
SBP (mmHg)	126.0 ± 11.0	117.6 ± 10.2	124.8 ± 10.6	116.0 ± 7.8	0.335	0.001	0.834
DBP (mmHg)	77.9 ± 7.2	74.2 ± 6.4	76.5 ± 7.0	72.6 ± 5.7	0.128	0.001	0.875
SCT (s)	2.9 ± 0.4	2.7 ± 0.4	3.5 ± 0.5	3.0 ± 0.4	0.001	0.001	0.112
SPPB score	11.9 ± 0.4	12.0 ± 0.1	11.9 ± 0.2	12.0 ± 0	0.124	0.027	0.465
PPT score	25.5 ± 1.6	26.6 ± 1.2	26.1 ± 1.3	27.1 ± 1.0	0.012	0.001	0.319

*All values are mean and standard deviation (SD). BMI, body mass index; BMI (SDS), body mass index standard deviation score; SBP, systolic blood pressure; DBP, diastolic blood pressure; HR, heart rate; SCT, stair climbing test; SPPB, short physical performance battery; PPT, physical performance test. Gender (G) and Time (T); Gender × time interaction (G × T).*

**FIGURE 1:**
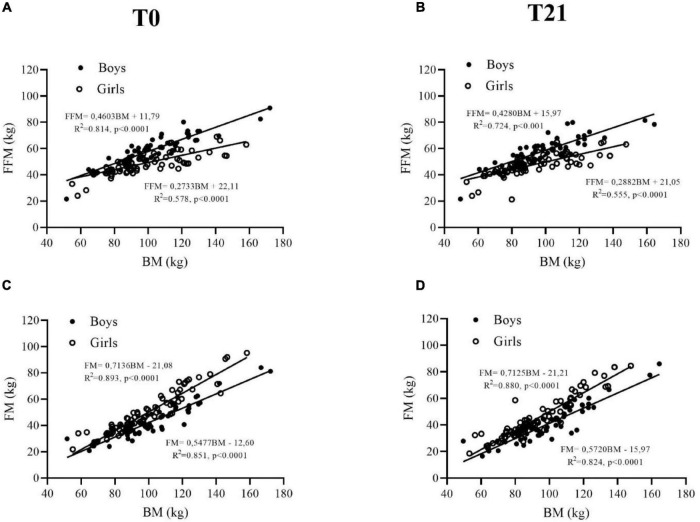
Fat-free mass (FFM) **(A,B)** and fat mass (FM) **(C,D)** are plotted as a function of body mass in adolescent obese boys (closed symbols) and girls (open symbols) before (T0) and after (T21) the 3-week BWRP.

At T0, FFM (kg) and FFM (%) were 22 and 13% higher in boys than in girls, respectively (*p* < 0.001, Table 1 and Figure 1A). Regarding FM (kg), no difference was found between boys and girls (Table 1 and Figure 1C), while FM expressed as a percentage was ∼15% (*p* < 0.001, Table 1) lower in boys than in girls. At T21, FFM (kg) decreased by 3% (*p* = 0.013, Table 1) in boys only (Figure 1B), while FFM (%) increased by ∼3 and ∼2% (*p* < 0.005, Table 1) in boys and girls, respectively. In contrast, FM (kg) decreased by ∼9 and ∼7% in boys and girls, respectively (*p* < 0.001, Table 1 and Figure 1D), with a smaller decrease when FM was expressed as a percentage (∼3 and ∼2% in boys and girls, respectively, *p* = 0.017, Table 1).

At T0, systolic BP and diastolic BP were not significantly different between boys and girls (Table 1). At T21, systolic BP and diastolic BP decreased by ∼7% in both genders (*p* < 0.001, Table 1).

#### Physical Capabilities

At T0, SCT and PPT scores were ∼11 and ∼3% lower in boys than in girls, respectively (*p* < 0.05, Table 1), while SPPB did not differ significantly between boys and girls (Table 1). At T21, SCT decreased by ∼5 and ∼7% (*p* < 0.001) in boys and girls, respectively. While PPT score increased by ∼4% (*p* < 0.001, Table 1) in both boys and girls. The SPPB score did not change significantly in both genders (Table 1).

Changes in FM (ΔFM) (kg) were not related to changes in SCT (ΔSCT), SPPB (ΔSPPB), PPT (ΔPPT, score), systolic BP (ΔSBP), and diastolic BP (ΔDBP) in both boys and girls (Table 2).

**TABLE 2:** Linear regression between changes in Fat Mass (ΔFM) (kg) in adolescents with obesity and possible predictors.

	Boys (*n*: 56)		Girls (*n*: 83)	
	R^2^	*p*-Value	R^2^	*p*-Value
ΔSCT (s)	0.007	0.523	0.008	0.416
ΔSPPB (score)	0.057	0.076	0.032	0.103
ΔPPT (score)	0.002	0.719	0.015	0.271
ΔSBP (mmHg)	0.046	0.110	0.008	0.415
ΔDBP (mmHg)	0.013	0.523	0.043	0.059

*SCT, stair climbing test; SPPB, short physical performance battery; PPT, physical performance test; SBP, systolic blood pressure; DBP, diastolic blood pressure; Δ is the difference of the values between the completing and the beginning of the weight reduction program.*

### Effects of the 3-Week BWRP on Adults

#### Anthropometric Characteristics and Body Composition

At T0, mean age and BMI did not differ significantly between males and females (Table 3), whereas stature and BM were significantly higher in males than females by ∼7 and ∼10%, respectively (*p* < 0.005, Table 3). At T21, BM and BMI decreased by ∼5 and ∼4% (*p* < 0.001, Table 3) in males and females, respectively.

**TABLE 3:** Physical characteristics of adults with obesity before (T0) and after (T21) the 3-week BWRP.

	Males (*n*: 27)		Females (*n*: 44)		*P*		
	T0	T21	T0	T21	G	T	G × T
Age (year)	56.8 ± 11.2		50.9 ± 15.1		0.060		
Stature (m)	1.71 ± 0.06		1.60 ± 0.06		0.001		
Body mass (kg)	125.8 ± 16.0	119.0 ± 14.9	113.9 ± 16.5	109.2 ± 15.7	0.007	0.001	0.001
BMI (kg/m^2^)	43.1 ± 3.6	40.8 ± 3.3	44.9 ± 5.8	43.0 ± 6.0	0.101	0.001	0.001
Fat-free mass (kg)	66.5 ± 7.4	65.3 ± 6.6	49.6 ± 4.1	48.8 ± 3.5	0.001	0.007	0.650
Fat-free mass (%)	53.1 ± 4.3	55.2 ± 4.3	44.1 ± 5.1	45.2 ± 4.8	0.001	0.001	0.128
Fat mass (kg)	59.3 ± 11.3	53.6 ± 10.8	64.3 ± 14.6	60.4 ± 13.6	0.067	0.001	0.046
Fat Mass (%)	46.9 ± 4.3	44.8 ± 4.3	55.9 ± 5.1	54.7 ± 4.8	0.001	0.001	0.129
SBP (mmHg)	143.1 ± 12.3	129.3 ± 11.7	133.5 ± 12.7	127.6 ± 10.0	0.015	0.001	0.018
DBP (mmHg)	85.2 ± 8.6	80.0 ± 6.2	82.2 ± 8.8	80.1 ± 5.1	0.285	0.003	0.183
SCT (s)	4.3 ± 1.2	3.8 ± 0.9	6.5 ± 5.3	5.6 ± 3.9	0.035	0.001	0.230
SPPB (score)	11.1 ± 1.3	11.8 ± 0.5	10.2 ± 2.5	11.2 ± 1.6	0.085	<0.001	0.473
PPT (score)	24.4 ± 2.4	26.7 ± 1.2	24.2 ± 4.4	26.4 ± 3.4	0.787	<0.001	0.834

*All values are mean and standard deviation (SD). BMI, body mass index; SBP, systolic blood pressure; DBP, diastolic blood pressure; HR, heart rate; SCT, stair climbing test; SPPB, short physical performance battery; PPT, physical performance test.*

*Gender (G) and Time (T); Gender × time interaction (G × T).*

**FIGURE 2:**
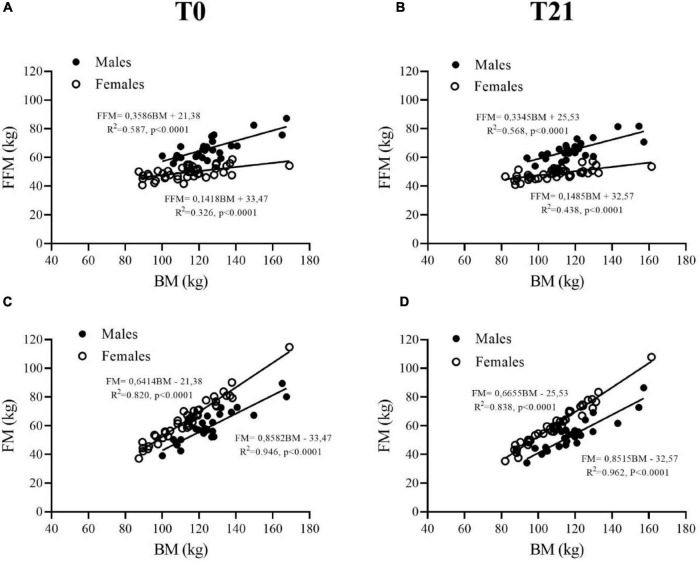
Fat free mass (FFM) **(A,B)** and fat mass (FM) **(C,D)** are plotted as a function of body mass (BM) in adult obese males (closed symbols) and females (open symbols) before (T0) and after (T21) of the 3-week BWRP.

At T0, FFM (kg) and FFM (%) were higher in males than females (+25% and +17%, respectively, *p* < 0.001), as shown in Table 3 and Figure 2A. FM (kg) was not significantly different between males and females (*p* = 0.240, Table 3 and Figure 2C), whereas males showed lower FM (%) than females (-16%, *p* = 0.001, Table 3). At T21, FFM (kg) decreased by ∼2% in males and females with significant time interaction (*p* = 0.007) (Table 3 and Figure 2B), while FFM (%) increased in males (∼4%, *p* < 0.001) and females (∼3%, *p* = 0.012) (Table 3). FM (kg) decreased by ∼10 and ∼6% in males and females, respectively (*p* < 0.001, Table 3 and Figure 2D), although a decrease was also observed when FM was expressed as a percentage, in both males (∼5%, *p* = 0.002) and females (∼2%, *p* = 0.012) (Table 3).

At T0, systolic BP at rest was greater in males (+7%, *p* = 0.001, Table 3) than in females, while diastolic BP did not differ significantly between the two genders. At T21, systolic BP at rest reduced significantly in both males (∼10%, *p* = 0.001) and females (∼5%, *p* = 0.010, Table 3), while diastolic BP reduced significantly by ∼6% in males only (*p* = 0.012, Table 3).

### Physical Capabilities

At baseline, SCT was lower in males than females by ∼5% (*p* = 0.042), whereas SPPB and PPT scores were not significantly different between the two genders (Table 3).

At T21, SCT decreased significantly only in females by ∼16% (*p* < 0.001, Table 3). SPPB score increased by ∼7 and ∼10% (*p* < 0.010, Table 3) in males and females, respectively. Similarly, PPT score increased by mean 9% (*p* < 0.001, Table 3) in the two genders.

Changes in FM (ΔFM) (kg) were inversely related to changes in both systolic BP (ΔSBP) (R^2^ = 0.242, *p* = 0.009; Table 4) and diastolic BP (ΔDBP) (R^2^ = 0.203, *p* = 0.009; Table 4) in males. In obese females, changes in FM (ΔFM) (kg) were inversely related to changes in both SPPB (ΔSPPB) (R^2^ = 0.345, *p* < 0.001; Table 4) and PPT (ΔPPT) (R^2^ = 0.187, *p* = 0.003; Table 4).

**TABLE 4:** Linear regression between changes in Fat Mass (ΔFM) (kg) in adults with obesity and possible predictors.

	Males (*n*: 27)		Females (*n*: 44)	
	R^2^	*p*-Value	R^2^	*p*-Value
ΔSCT (s)	0.002	0.942	0.030	0.278
ΔSPPB (score)	0.002	0.837	0.346	0.001
ΔPPT (score)	0.002	0.816	0.187	0.003
ΔSBP (mmHg)	0.242	0.009	0.001	0.802
ΔDBP (mmHg)	0.203	0.018	0.025	0.297

*SCT, stair climbing test; SPPB, short physical performance battery; PPT, physical performance test; SBP, systolic blood pressure; DBP, diastolic blood pressure; Δ is the difference of the values between the completing and the beginning of the weight reduction program.*

## Discussion

The present study shows that a 3-week inpatient multidisciplinary BWRP, which includes physical training, moderate energy restriction, nutrition education, and psychological counseling, determines in adolescents and adults with obesity: (1) a significant reduction in BM and FM with a slight decrease in FFM, (2) improvements in SCT time, SPPB score in adults and PPT score in both groups, and (3) a positive relationship between reduction in FM and reduction in systolic BP and diastolic BP in obese male adults.

Our intervention was effective in reducing BM and FM for both adolescents and adults with obesity, in agreement with previous results reported in our laboratory after 3-week inpatient BWRP for patients aged 8–17 years (38, 39) and even for older obese male and females aged 61–75 year (40), confirming that body composition can be improved in obese individuals at any age. However, during the short-term BWRP adolescents and adults with obesity have shown a slight reduction in FFM, probably because no specific strength training was considered in this first phase of metabolic rehabilitation of patients with obesity, as previously observed (41).

After a 3-week of BWRP, SCT time was reduced after the BWRP in adolescents and in female, evaluated with the modified Margaria test (42) previously used to assess lower limb muscle power in obese subjects (43). Although there was no direct correlation between the BM reduction and the improvement in SCT time, as previously observed by our research group (17), it seems plausible to hypothesize that the reduction of BM may be associated with reduced body inertia (44) and the reduction of intramyocellular lipids may have played a role in the improvement of lower limb strength evaluated in our study by SCT (45). SPPB is a test useful for assessing lower limb functionality (static balance, 4 m walking and lower limb strength) (46). In our study, although SPPB scores were at the upper limit of normality (i.e., in the range of 11–12 points) (34), improvements of ∼0.8 and ∼1 points were found for male and female obese adults after 3-week BWRP, whereas smaller improvements, albeit statistically significant, were found in obese adolescents. In this regard, our findings are relevant because multidisciplinary 3-week BWRP appears to positively affect lower limb functionality in tasks such as walking and in those requiring getting up and down from a chair, which are typically impaired in obese patients (47–49), thus contributing to improve their quality of life. PPT is useful in assessing functional capabilities as it mimics ADL (36, 50–52). Previous studies showed that obese subjects had perceived difficulty in performing physical exercise and ADL (13–16). In the present study, the PPT score increased in both obese adolescents and adults, thus suggesting that a structured exercise program administered during a 3-week BWRP can improve the ability to perform ADL. Our findings are consistent with those recently reported by Wilson et al. (53), showing that regular physical activity and moderate energy restriction have positive effects on physical performance and quality of life. Furthermore, the improvements in SPPB and PPT scores in adult obese females were positively correlated with FM loss. At T0, mean SPPB and PPT scores in our obese females were lower than 11 and 28, respectively, and these two scores are considered signs of poor lower limb functionality (54, 55). Several factors have been considered to explain lower limb physical performance in females compared to males, such as lower muscle strength (56), higher body fat (56), lower muscle mass (57), and greater muscle fat infiltration (58) with the greatest adipose tissue thickness in the lower limbs (59).

Finally, reduction of FM was positively related to a reduction in systolic BP and diastolic BP only in obese males. Indeed, visceral adiposity is generally higher in obese male adults than in female counterparts (45), and visceral fat has been linked to the secretion of adipocytokines, that contribute to the development and progression of hypertension (60). Thus, BWRP has proven to be effective in improvement cardio metabolic profile of obese males (61). However, since we did not directly measured visceral adiposity, this missing information can be considered a limitation of the present study. Future studies involving BWRP in the hospital environment should measure visceral adiposity not only in relation to cardiovascular risk profile, but also as a marker of quality of life improvement in adolescents and adults with obesity.

## Conclusion

In conclusion, our study confirms that a 3-week inpatient multidisciplinary BWRP, even if it only detects a 4–5% reduction in BM, is able to induce a significant reduction in FM, improvements in lower limb functionality and lower limb muscle power, and improvements in performing ADL, all of which are positive changes highly relevant to improve quality of life of adolescents and adults with obesity.

## Data Availability Statement

The original contributions presented in the study are included in the article/supplementary material, further inquiries can be directed to the corresponding author.

## Ethics Statement

The studies involving human participants were reviewed and approved by Ethical Committee of the Istituto Auxologico Italiano (Milan, Italy). Written informed consent to participate in this study was provided by the participants’ legal guardian/next of kin.

## Author Contributions

AS, SL, MD’A, and FV: conceptualization. AS, MD’A, SL, FV, GT, and RD: data curation. FV and MD’A: formal analysis. AS: funding acquisition, project administration, and supervision. GT and RD: investigation. MD’A: writing original draft. AS, MD’A, SL, and FV: writing, review and editing. All authors have read and agreed to the published version of the manuscript.

## Conflict of Interest

The authors declare that the research was conducted in the absence of any commercial or financial relationships that could be construed as a potential conflict of interest.

## Publisher’s Note

All claims expressed in this article are solely those of the authors and do not necessarily represent those of their affiliated organizations, or those of the publisher, the editors and the reviewers. Any product that may be evaluated in this article, or claim that may be made by its manufacturer, is not guaranteed or endorsed by the publisher.

## Abbreviations

ADL: activities of daily living
BM: body mass
BMI: body mass index
BMI (SDS): body mass index standard deviation score
BP: blood pressure
BWRP: body weight-reduction programs
DBP: diastolic blood pressure
HR: heart rate
PPT: physical performance test
SBP: systolic blood pressure
SCT: stair climbing test
SPPB: short physical performance battery.
