# Corrigendum: Effects of a 3-week inpatient multidisciplinary body weight reduction program on body composition and physical capabilities in adolescents and adults with obesity

**DOI:** 10.3389/fnut.2022.1003940

**Published:** 2022-08-18

**Authors:** Stefano Lazzer, Mattia D'Alleva, Filippo Vaccari, Gabriella Tringali, Roberta De Micheli, Alessandro Sartorio

**Affiliations:** ^1^Department of Medicine, University of Udine, Udine, Italy; ^2^School of Sport Sciences, University of Udine, Udine, Italy; ^3^Experimental Laboratory for Auxo-Endocrinological Research, Istituto Auxologico Italiano, Scientific Institute for Hospitalization and Care (IRCCS), Piancavallo, Italy; ^4^Division of Auxology, Istituto Auxologico Italiano, Scientific Institute for Hospitalization and Care (IRCCS), Piancavallo, Italy

**Keywords:** physical capabilities, body composition, adolescents, adults, obesity, physical activity

A correction has been made to **Funding**. The correct Funding statement is:

“Research funded by the Italian Ministry of Health.”

The authors apologize for this error and state that this does not change the scientific conclusions of the article in any way. The original article has been updated.

## Publisher's note

All claims expressed in this article are solely those of the authors and do not necessarily represent those of their affiliated organizations, or those of the publisher, the editors and the reviewers. Any product that may be evaluated in this article, or claim that may be made by its manufacturer, is not guaranteed or endorsed by the publisher.

